# Removal of schwannoma from the psoas muscle with intraoperative neurophysiological monitoring: A case report

**DOI:** 10.1097/MD.0000000000037244

**Published:** 2024-02-16

**Authors:** Na Yoon Yoo, Hyoung Seop Kim, Joong Won Yang, Dougho Park

**Affiliations:** a Department of Physical Medicine and Rehabilitation, National Health Insurance Service Ilsan Hospital, Goyang, South Korea; b Department of Neurosurgery, Pohang Stroke and Spine Hospital, Pohang, South Korea; c Department of Rehabilitation Medicine, Pohang Stroke and Spine Hospital, Pohang, South Korea.

**Keywords:** intraoperative neurophysiological monitoring, lumbosacral plexus, psoas muscle, Schwannoma

## Abstract

**Rationale:**

The incidence of a schwannoma within the psoas muscle is rare, and only a few cases have been reported. The surgical approach to removing schwannomas present in the psoas muscle is challenging because of its anatomical proximity to the lumbar plexus.

**Patient concerns:**

A 31-year-old man experienced right lower back pain and anterolateral thigh numbness for 2 months.

**Diagnosis:**

Magnetic resonance imaging of the patient’s lumbar spine revealed a mass lesion, which was radiologically diagnosed as a well-demarcated schwannoma.

**Interventions:**

The patient underwent surgery for excision of the schwannoma in the right psoas muscle at the second to fourth lumbar vertebrae levels. During surgery, intraoperative neurophysiological monitoring modalities, free-running and triggered electromyography and evoked potentials, from the target muscles were recorded.

**Outcomes:**

There was no neurotonic discharge corresponding to neuronal injury. Compound motor nerve action potential was detected in the triggered electromyography of muscles around the medial margin of the tumor. However, direct integration of the motor nerve was not observed in the intra-tumor region.

**Lessons:**

We report that schwannoma removal in the psoas muscle, which is adjacent to the lumbar plexus, can be safely performed using intraoperative neurophysiological monitoring.

## 1. Introduction

Schwannoma is a tumor found in the nervous system and is mostly benign. Retroperitoneal schwannomas are rare and account for <3% of all schwannomas.^[1,2]^ Moreover, the incidence of a schwannoma within the psoas muscle is a rare phenomenon; few cases have been reported.^[3]^ The surgical approach to removing a schwannoma in the psoas muscle is challenging because of the potential injury that can be caused to the lumbar plexus due to its anatomical proximity.

Real-time detection of nerve damage during surgery is possible using intraoperative neurophysiological monitoring (IONM).^[4]^ The functional integrity of nerves can be confirmed, even in surgeries involving the possibility of a peripheral nerve injury, by using the direct nerve stimulation method within the surgical field.^[5]^ In this case report, we describe the safe removal of a schwannoma in the psoas muscle using IONM.

## 2. Case presentation

### 2.1. Patient information

The patient was a 31-year-old man with a complaint of right lower back pain and anterolateral thigh numbness for 2 months. He had no other comorbidities or family history. Written, fully informed consent for treatment and tests was obtained from the patient. Moreover, written informed consent for publishing the medical records and clinical data involved in this case report was obtained from the patient.

### 2.2. Preoperative evaluations

The patient complained of only unilateral sensory symptoms and exhibited intact motor powers in the bilateral lower extremities. The balance function was also intact with a Berg balance scale score of 56 points.

**Figure 1.**
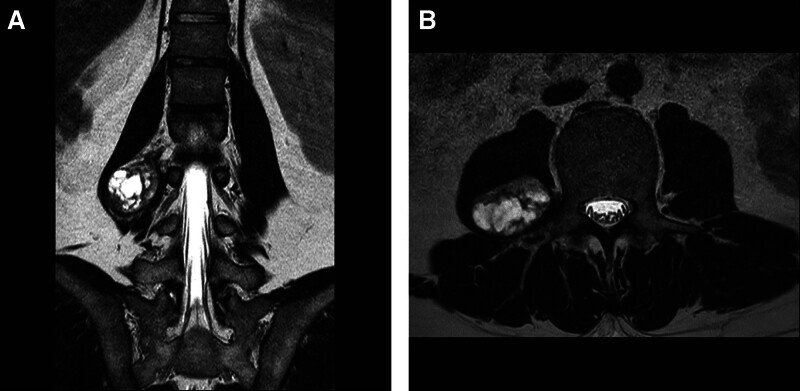
Preoperative magnetic resonance imaging (MRI) findings. A well-demarcated mass lesion in the right psoas muscle was identified in the (A) coronal and (B) axial views of MRI.

Magnetic resonance imaging of the patient’s lumbar spine revealed a lesion measuring 4 cm × 3.1 cm × 5.2 cm in the right psoas muscle at the level of the second to fourth lumbar vertebrae. The lesion was radiologically diagnosed as a well-demarcated schwannoma (Fig. 1).

The nerve conduction study and needle electromyography (EMG) performed before surgery revealed no root or peripheral nerve lesions in the right lower extremity. Somatosensory evoked potential of the lateral femoral cutaneous nerve was normal.

### 2.3. Surgical approach

The patient was positioned in the right decubitus position. While surgery was performed under general anesthesia, which was administered in the form of total intravenous anesthesia, neuromuscular blockade was not used during the surgery since evoked potentials and EMG waves in the designated muscles had to be recorded.

The C-arm was used to confirm the level of the third to fourth lumbar vertebrae, which was the index level. The surgeon (J.W.Y) dissected the abdominal muscle layer after making an oblique skin incision. After performing a finger dissection of the extraperitoneal fat layer using peanut balls, the surgeon confirmed the presence of the psoas and quadratus lumborum muscles. Subsequently, he dissected the right psoas muscle fiber above the tumor and found the mass. He then performed circumferential dissection around the tumor and piecemeal removal of the tumor. Finally, he separated the proximal originating tumor from the psoas muscle.

### 2.4. IONM findings

**Figure 2.**
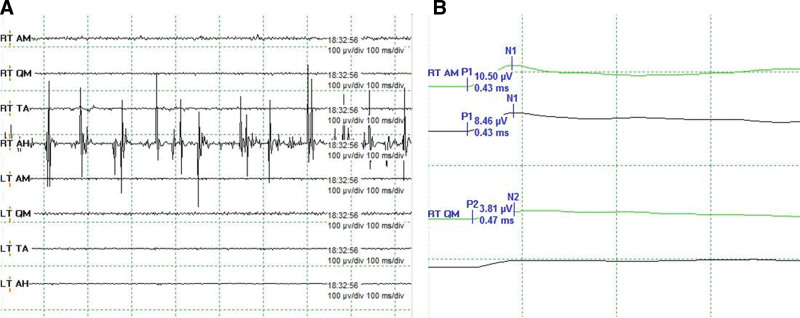
Intraoperative neurophysiological monitoring findings. (A) Mild burst patterns were observed in the right abductor hallucis muscles. (B) Direct stimulation with maximal stimulation (25 mA) evoked compound motor nerve action potentials in the right adductor magnus and vastus medialis muscles.

During surgery, free-running EMG, evoked EMG, and motor evoked potential were recorded from the target muscles: the vastus medialis, adductor magnus, tibialis anterior, and abductor hallucis muscles. Following a visual identification of the tumor, triggered EMG (direct stimulation) was monitored to confirm connectivity and proximity with the lumbar plexus during tumor resection. Mapping was conducted with a monopolar ball tip probe, and stimulation was initiated at intervals of 0.1 ms from a voltage of 4 mA, which was increased to 25 mA. Free-running EMG of the right abductor hallucis demonstrated a mild burst pattern (Fig. 2A). However, no neurotonic discharge corresponding to neuronal injury was observed. There was no direct integration of the motor nerve in the intra-tumor region; however, a compound motor nerve action potential was observed in the trigger EMG of the right adductor magnus and vastus medialis around the medial margin of the tumor (Fig. 2B). The somatosensory evoked potential of the right lateral femora cutaneous nerve, which was recorded, indicated no change in amplitude and latency during the operation.

### 2.5. Postoperative evaluations

The excised mass was histologically confirmed to be a schwannoma (neurilemmoma). Immediately after surgery, the patient complained of right back pain and numbness of the anterolateral thigh. Electromyography studies performed after the surgery did not reveal a root or peripheral nerve lesion.

The symptoms of anterolateral thigh numbness and right back pain improved 1 month after surgery. The muscle strength of both lower extremities remained intact. Six months following the surgery, the patient was free of all symptoms.

## 3. Discussion

This case involved the safe removal of a schwannoma that was adjacent to the lumbar plexus using IONM. Mass removal adjacent to the neural plexus can be challenging because nerve damage during peripheral nerve surgery can lead to severe complications such as motor weakness. This case confirmed that IONM is an effective and safe tool for detecting nerve damage during peripheral nerve surgery.

IONM is generally used in surgeries that carry a risk of injury to the central nervous system during the procedure, such as brain tumor removal, cerebrovascular surgery, or spinal cord surgery.^[6]^ The main modalities of IONM are evoked potentials, spontaneous EMG, and triggered EMG.^[7]^ These modalities should be comprehensively interpreted to detect neural injury and determine the state of the patient. Spontaneous EMG represents the electric signals from the muscles recorded passively, whereas triggered EMG represents the signals induced by the direct stimulation of nerve or hardware; therefore, they can provide immediate and reliable information about any nerve injury to the surgeon.^[8]^

Image findings can largely enable surgeons to detect and diagnose lesions. However, using other modalities such as electrophysiological results, including IONM, in combination can enable the provision of additional data on the functional integrity of nerves.^[9]^ This allows surgeons to perform the removal of the mass adjacent to the lumbar plexus with greater assurance, thereby improving the prognosis of the surgery. Several cases of peripheral nerve surgery performed using IONM have been reported. Kravtsov et al^[10]^ reported a case of neurinoma of the fifth lumbar nerve root that was surgically removed using endoscopy along with IONM; although motor weakness of the great toe extensor persisted, the patient’s pain of the L5 dermatome, which was present prior to the surgery, improved from a visual analog scale score of 7 to 2. In another case involving the surgical release of a ganglion cyst that was compressing the posterior interosseous nerve, resulting in symptoms of wrist and finger drop before the surgery, which was performed under IONM^[11]^; partial recovery of wrist and finger extensor power was observed after the surgery. Patel et al^[12]^ demonstrated that IONM could minimize the minor and major neural injuries during revision shoulder arthroplasty surgeries. The case in this study also demonstrated that using IONM could assure the safety of the patient while performing the excision of a mass adjacent to the neural structure, thereby corroborating the findings of the aforementioned studies.

In our patient, compound motor nerve action potential was induced by maximal intensity stimulation in triggered EMG and detected at the medial margin of the tumor; however, it was not induced in the intra-tumor region. Thus, it was concluded that the tumor did not directly interface with the lumbar plexus. Transient bursts can be induced by mild traction in free-running EMG but are reversible.^[13]^ Therefore, it was concluded that there was no traction or irritation that could have led to irreversible nerve injury.

The patient only complained of sensory symptoms. However, normal results were obtained during the preoperative and postoperative nerve conduction studies, and the somatosensory evoked potentials measured during surgery were also normal. Therefore, the sensory symptoms could be attributed to the mass effect rather than the direct integration of the schwannoma with the sensory fiber. The sensory symptoms improved after the excision of the tumor, likely because of decompression effects.

There were some limitations in this study. This study includes only 1 case, and further research is needed to validate our results. Additionally, comparisons of cases conducted using the IONM modality with and without neural insult occurrence are required.

In conclusion, this case confirms that removal of schwannoma in the psoas muscle, which is adjacent to the lumbar plexus, can be performed safely using IONM. In peripheral nerve surgery, IONM enabled safe tumor resection without complications.

## Author contributions

**Investigation:** Na Yoon Yoo, Hyoung Seop Kim, Joong Won Yang, Dougho Park.

**Supervision:** Hyoung Seop Kim, Joong Won Yang.

**Visualization:** Dougho Park.

**Writing – original draft:** Na Yoon Yoo, Joong Won Yang, Dougho Park.

**Writing – review & editing:** Hyoung Seop Kim, Dougho Park.
